# The Relation between Body Mass Index and Retinal Photoreceptor Morphology and Microvascular Changes Measured with Adaptive Optics (rtx1) High-Resolution Imaging

**DOI:** 10.1155/2021/6642059

**Published:** 2021-02-26

**Authors:** Anna Zaleska-Żmijewska, Zbigniew Wawrzyniak, Magdalena Kupis, Jacek P. Szaflik

**Affiliations:** ^1^Department of Ophthalmology, SPKSO Ophthalmic Hospital, Medical University of Warsaw, Warsaw, Poland; ^2^Faculty of Electronics and Information Technology, Warsaw University of Technology, Warsaw, Poland

## Abstract

**Background:**

Overweight and obese patients are at risk for diabetes, cardiovascular disorders, and microvascular complications. The rtx1^TM^ (Imagine Eyes, France) is a microscope that allows near histological visualizations of cones and retinal microcirculation.

**Objective:**

This study analysed the cones and retinal microvascular changes in a group of 47 healthy women with different BMI values. Participants were divided into 2 groups: the BMI group (28 women with BMI >/25) and the control group (19 lean women with BMI <25).

**Results:**

The lumen and diameter of retinal arteries were not significantly different between groups. There were significant differences in the thickness of arteriole walls. The WLR and WCSA values differed significantly between the control and BMI groups (for WLR 0.25 ± 0.03 vs. 0.29 ± 0.03, *p* < 0.001; for WCSA 4136.7 ± 1140.0 vs. 5217.3 ± 944.0, respectively, *p* < 0.001). In healthy eyes, cone density and morphology were not affected by weight.

**Conclusions:**

Retinal image analysis with rtx1 offers a novel noninvasive measurement of early changes in retinal vasculature that are not detectable during routine clinical examination. Abnormalities of retinal arterioles found by rtx1™examination should be considered as a strong risk factor for cardiovascular changes resulting from overweight and obesity.

## 1. Introduction

The impact of obesity on general health and increased risk of cardiovascular diseases (CVD) is well known [1–6]. The relationship between body mass index (BMI), CVD risk factors, and vascular disease endpoints was prospectively examined in Framingham Heart Study participants, who were followed for up to 44 years [1]. People with obesity more often develop coronary heart disease and large-vessel atherosclerosis in the carotid and femoral arteries [2, 4–6]. The increased carotid intima-media thickness (IMT), which is one of the early markers of subclinical atherosclerosis and may predict future myocardial infarction and stroke, was found in young, healthy women, with no known cardiovascular disease but with obesity [7]. There is some evidence that, even in healthy individuals, obesity is associated with impaired microvascular function and increased risk of hypertension and insulin resistance [8–12].

The retina is an easily accessible part of the microcirculation and in vivo evaluation of microvascular changes due to different disorders can be done by examining the eye. The development of diagnostic tools for retinal microvasculature has been difficult because of its location in the posterior segment of the eye and very small size (200–300 *μ*m in thickness) [13, 14]. New diagnostic imaging methods have gradually been introduced, including colour fundus photography, fluorescein angiography, scanning laser ophthalmoscopy, and optical coherence tomography (OCT) [14]. Digital retinal photography and new imaging technologies now allow more precise noninvasive visualization of subtle retinal changes. The high reliability of retinal photography has been proved in the Atherosclerosis Risk in Communities Study (ARIC) [15].

Advances in retinal imaging devices and the development of more sophisticated optical systems have made it possible to capture enface images of photoreceptors and microcirculation. Adaptive optics (AO) is a noninvasive method for visualizing microcirculation and retinal structures with resolution comparable to those achieved in histological studies [16–18]. The rtx1^TM^ (Imagine Eyes, Orsay, France) is a microscope that uses AO technology. The AO imaging system corrects aberrations arising from various refractive surfaces within the eye. The rtx1 microscope includes image acquisition and object recognition software for image analysis oriented towards cones and vessels [16].

The available international literature offers several publications that examine findings using the rtx1 in healthy patients [18–22]. To our knowledge, there are no publications based on rtx1 measurements regarding changes in the retinal photoreceptors and retinal arterioles depending on the body mass index (BMI).

This study therefore analysed cone parameters and retinal microvascular changes in a group of healthy women with different BMI values.

## 2. Methods

Retinal examinations with the rtx1 device were conducted between May and July 2018 at the Department of Ophthalmology, Second Faculty of Medicine, the Medical University of Warsaw, located in the Ophthalmic University Hospital in Warsaw. The study protocol was approved by the Bioethical Commission of the Medical University of Warsaw. Each patient received both oral and written information explaining the objective and design of the study, the operating principles of the device, and the examination procedure. In accordance with the Declaration of Helsinki, written informed consent was obtained from all subjects who participated in the study.

### 2.1. Eligibility Criteria

The study group consisted of adult (>18 years, white European) women, volunteers, with normal weight, overweight, or obesity with no known ocular and systemic diseases. Participants were recruited through online advertisements or were relatives or friends of other participants. All patients provided a medical history and current blood samples to verify inclusion and exclusion criteria. The blood tests were collected two days before the retinal examinations with rtx1.

The inclusion criteria for the study group were female gender and, for normal weight, BMI <24.99 kg/m^2^; for overweight, BMI >25 kg/m^2^ <29.9 kg/m^2^; for obesity, BMI >30 kg/m^2^. Besides the increased values of BMI participants, participants had no known diseases related to obesity and were not taking any treatment.

The exclusion criteria included the following: diagnosed and treated prediabetic syndrome, diabetes mellitus, systemic hypertension, hyperlipidaemia, and cigarette smoking, as well as the use of steroids, oral contraception pills, or any anabolic agents.

Hypertension was defined as systolic blood pressure >/140 mmHg, diastolic blood pressure >/90 mmHg, or self-reported physician diagnosis of hypertension or current use of antihypertensive medications. Diabetes mellitus was defined as fasting plasma glucose (FPG) test >/126 mg/dl or if the patient was taking medication for diabetes. There was no possibility of performing a 2-hour oral glucose tolerance test (OGTT) during the screening visit, so only patients with already diagnosed impaired fasting glucose (IFG) were excluded from the study. BMI was calculated as weight in kilograms divided by the square of height in meters.

The ophthalmic exclusion criteria were refractive errors for myopia >6 dioptre or astigmatism >2.50 dioptre cylindrical, the presence of media opacities, a scar in the fovea, and no central fixation.

**Figure 1:**
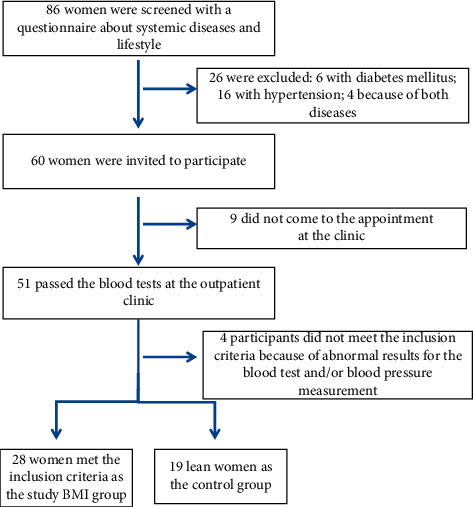
Flowchart diagram of participant sampling, recruitment, and exclusion criteria.

Eligibility for study participation was confirmed by comprehensive ocular examination. Participant sampling, recruitment, and exclusion are described in the flowchart diagram (Figure 1).

### 2.2. Patients Characteristics

Initially, a group of 86 women volunteers were asked to complete a questionnaire about lifestyle risk factors and medical history. Of this group screened, 26 were excluded due to systemic diseases, which were currently treated (diabetes mellitus or impaired glucose tolerance, 6; hypertension, 16; and both diseases in 4 cases); 60 women were invited to participate in the study, and 51 came to the Outpatient Clinic for blood tests. Based on the results of the blood tests, we had to exclude a further 4 women. The final group of 47 participants was divided into two groups. The study group consisted of 28 women with BMI >25 and without any systemic diseases treated up to the time when the study procedures were performed (BMI group); 11 of these were overweight and 16 were obese. The control group consisted of 19 lean women with BMI <25 (control group; Figure 1).

### 2.3. Image Acquisition with the rtx1^TM^adaptive Optics (AO) Camera

An AO retinal camera (rtx1^TM^; Imagine Eyes, Orsay, France) was used to acquire images of parafoveal cones and retinal arterioles in all participants. The imaging device is composed of 3 main components: high-resolution fundus camera, a Shack–Hartmann wavefront sensor, and a deformable mirror for real-time correction of the aberrations of the ocular wavefront. The rtx1™uses enface reflectance imaging with flashed, noncoherent near infrared illumination. Two computer programs are provided by the manufacturer to analyse the examination findings: AOdetect (for photoreceptor analysis) and AOdetectArtery (for retinal vasculature analysis).

All subjects also had noncontact ocular biometry using the IOL Master (Carl Zeiss Meditec AG, Hennigsdorf, Germany).

### 2.4. Protocol for rtx1 Examination and Evaluation Parameters

Each rtx1 examination captured scans of the 4 perifoveal areas of the retina, 2° (approximately 540–600 *μ*m) off the centre of the fovea (temporally, nasally, superiorly, and inferiorly) with a standardized 80 × 80 *μ*m sampling window size (fixed by the manufacturer in the new model). Most of the examinations did not require dilation of the pupils. In cases in which the width of the pupil was less than 4 mm, one drop of 1% tropicamide (Polfa Warszawa) was administered. None of the participants from the control group required pupil dilation and only 4 (14.3%) participants from the BMI group had the image acquisition after 1% tropicamide drop. Study participants were instructed to fixate on the yellow fixation cross in the camera. After finding the foveal reference point of the patient, eccentricities of 2° along the meridians were measured and used for further images analyses. In most cases, the acquisition of the best image of cones required at least 3 scans. In retinal arteries imaging, one scan was enough. It took approximately only 2–5 seconds to reach a single scan. The best sections of images captured were selected and analysed using the image processing and recognition software AOdetect and AOdetectArtery.

The analyses of the photoreceptors included the mean (±standard deviation) cone density per square millimetre of the retinal surface and the morphology of the cones in terms of the neighbourhood (Voronoi domain), regularity, and cones spacing. The arithmetic average of 3 measurements of the selected cones images was taken for statistical analysis.

In the analyses of retinal vessels, three measurements of the retinal arteriole with a size between 70 and 130 *μ*m in the temporal superior quadrant were taken. The arithmetic average of these 3 values was taken. Measurements of the total vessel diameter (VD), the two walls thickness (wall 1, wall 2), and the lumen diameter (LD) were recorded, and the wall-to-lumen ratio (WLR) and the cross-sectional area of the vascular wall (WCSA) were then automatically calculated with the dedicated software. Vessel diameter resulted from the single arteriolar wall (WT) plus vessel lumen (LD) and single arteriolar wall thickness (WT) : VD = WT + (WT + LD). WLR was calculated as WLR = 2·WT/LD. WCSA of the vascular wall was measured on the basis of vessel diameter and lumen diameter, and the value was obtained automatically from the AO artery detect software. We use automated cone and vessel parameters identification, in some cases with manual review of the artery profile.

### 2.5. Medical History of the BMI Group and the Control Group

The results of blood samples were analysed, including FPG, glycated haemoglobin A_1c_ (HbA_1c_), triglyceride (TG), total cholesterol, low-density lipoprotein cholesterol (LDL), high-density lipoprotein cholesterol (HDL), white blood cells (WBC), red blood cells (RBC), platelets, BMI, and the correlation between chosen parameters and cone density and retinal artery parameters.

### 2.6. Statistical Analyses

Descriptive analyses were conducted for all variables that were visually assessed for outliers. Kolmogorov–Smirnov and Shapiro–Wilk tests were used to determine if the parameters were normally distributed. Because the parameters were not normally distributed, we used the Wilcoxon signed rank test to compare data from the right and the left side and the Mann–Whitney *U* test to compare the BMI and control groups.

Simple and multiple linear regressions were performed to determine the effect of blood and serum lipid parameters and BMI on parameters measured using the rtx1. Potential modifiers were examined in stratified analysis. All probabilities quoted are 2-sided, and a significant *p* value was defined as <0.05. Statistically significant results obtained using *t* test and Mann–Witney *U* test are presented after Bonferroni correction for multiple comparisons [23].

The Pearson correlation coefficient (*r*) was calculated to examine the linear relationship between two continuous variables. Statistical analyses were generated with TIBCO™ Statistica™ 13.1 (data analysis software system), version 13 (http://www.tibco.com).

## 3. Results

The best-corrected visual acuity (BCVA) was 1.0 in both groups.

Age and axial length were not significantly different between groups. The mean (±standard deviation) age in the control group and the BMI group was 45.05 (±9.7) and 50.79 (±6.42), respectively (*p*=0.0654).

The mean axial length of the right eye (RE) in the control group was 23.12 ± 0.97 mm and that of the left eye (LE) was 23.12 ± 1.17 mm (Mann–Whitney *U* test, *p*=0.580). The mean axial length of the RE in the BMI group was 23.21 ± 0.99 mm and that of the left eye was 22.68 ± 1.02 mm (*pt* test, *p*=0.417). Because eyes did not differ in terms of axial lengths, only results obtained from right eyes were included in further analyses.

The characteristics of both groups are shown in Table 1.

The results of the blood test after Bonferroni correction (adjusted *p* value = 0.0071) presented in Table 2 are not significant. The results of serum lipid parameters revealed significant differences between groups in levels of HDL and TG after Bonferroni correction (adjusted *p* value = 0.0125). Compared with lean women, women with higher BMI levels had dyslipidaemia with significantly lower HDL values and higher TG. The mean FPG and LDL were slightly higher in the group with greater BMI, but the difference was not significant. The results of the blood and serum lipid parameters for both groups are presented in Tables 2 and 3.

Although all individuals were normotensive, systolic (SBP) and diastolic (DBP) blood pressure were significantly higher in women with higher BMI compared to the control group (SBP 138.1 ± 7.0 vs. 128.7 ± 2.3, *p*=0.02; DBP 83.4 ± 5.7 vs. 76.3 ± 1.5, *p*=0.03, respectively).

### 3.1. Cone Parameters

Cone density was significantly lower after Bonferroni correction and interphotoreceptor spacing higher in the BMI group compared to the control group at the 2° retinal eccentricities only in the temporal quadrant. In other locations, the cone density was comparable between groups, with a higher standard deviation in the control group. The results of cone density for all quadrants are presented in Table 4.

Cone packing regularity was assessed through analysis of the Voronoi domains. There were no significant differences in cone regularity after Bonferroni correction (adjusted *p* value = 0.0042) and the mean percentage of cones with hexagonal Voronoi tiles (*N*%6) in all analysed locations of the retina between both groups (Table 5).

### 3.2. Retinal Artery Parameters

The mean lumen and total diameter of the analysed retinal arteries were not significantly different between groups. The mean of both artery walls (wall1, wall2) were significantly thicker in the BMI group than in the control group (*p* < 0.001) after Bonferroni correction (adjusted *p* value = 0.0084). The mean WLR and WCSA values also differed significantly between the control and the BMI groups (for WLR 0.25 ± 0.03 vs 0.29 ± 0.03, *p* < 0.001; for WCSA 4136.7 ± 1140.0 vs. 5217.3 ± 944.0, respectively, *p* < 0.001) after Bonferroni correction (adjusted *p* value = 0.0084), as shown in Table 6.

**Figure 2:**
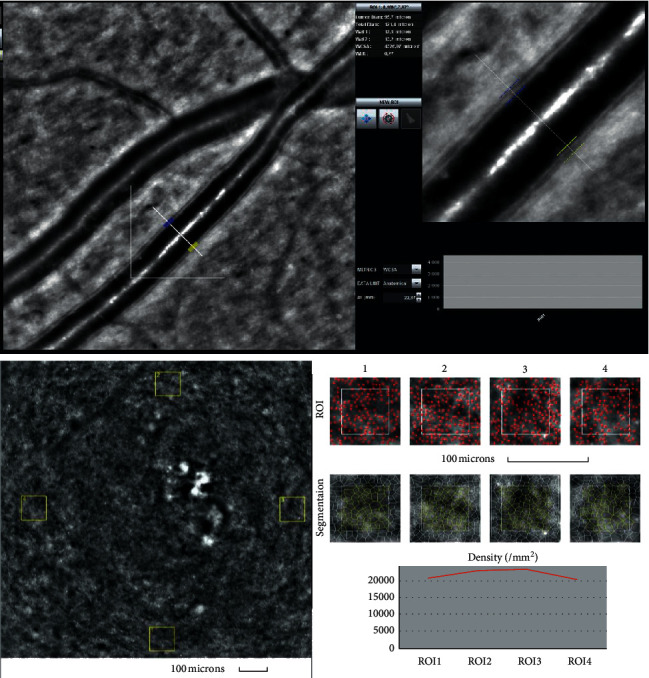
Image of the retinal artery (upper) and of four retinal cones squares windows (cones depicted in red and Voronoi triangulation) (lower) for a patient from the control group (IK) captured by rtx1 AO retinal camera. The following parameters are shown: in the artery analysis; Lumen: 96.7 *μ*m, Total diameter: 121.4 *μ*m, Wall_1: 12.5 *μ*m, Wall_2: 13.2 *μ*m, WLR- 0.27, WCSA- 4277.0 *μ*m^2^, AL- 22.87 mm; in the cone analysis: for different ROI (region of interest) squares: Regularity: 82.9–92.5%, Cone spacing: 7.21–7.71 *μ*m, Cone density: 20264–22831 cone/mm^2^.

**Figure 3:**
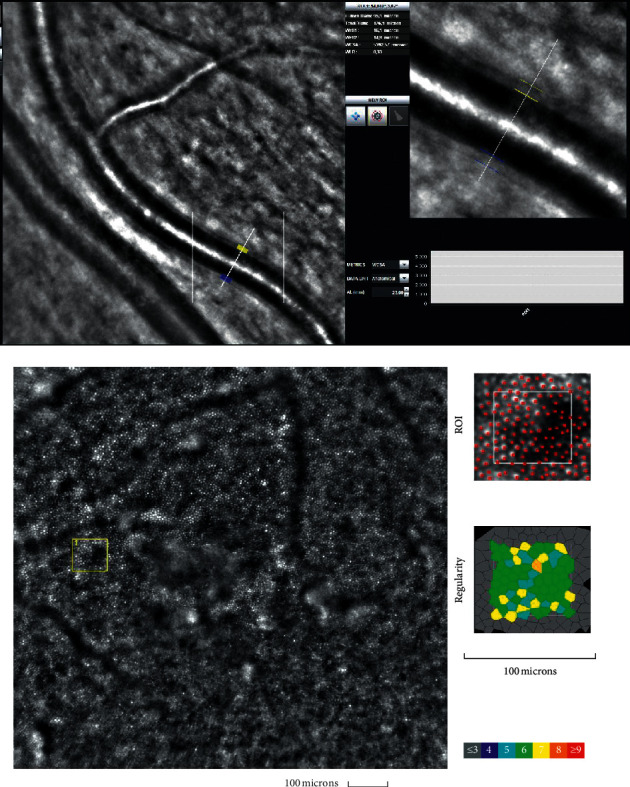
Image of the retinal artery (upper) and the cones mosaic window (cones depicted in red and Voronoi triangulation) (lower) for a BMI group patient (AR) captured by rtx1 AO retinal camera. The following parameters are shown: in the artery analysis; Lumen: 95.1 *μ*m, Total diameter: 126.1 *μ*m, Wall_1: 16.1 *μ*m, Wall_2: 14.9 *μ*m, WLR: 0.33, WCSA: 5302.6 *μ*m^2^, AL: 23.00 mm; in the cone analysis: for different ROI (region of interest) squares: Regularity: 98.6%, Cone spacing: 7.89 *μ*m, Cone density: 19636 cone/mm^2^.

Figures 2 and 3 present the analysis of the cones mosaic morphology and the retinal artery taken from an eye of a patient from the control group and from the BMI group, respectively. The image of the microvascular changes in an artery taken from an overweight woman is shown in Figure 3, with increased WLR, WCSA, and thickening of arteriole walls (Figures 2 and 3).

### 3.3. Correlation between Cones and Artery and Parameters

Adjustment WLR for diastolic blood pressure, lumen, and HbA1c revealed the association between increased BMI and increased wall-to-lumen ratio in linear regression analysis.

No correlations were found between cone parameters and any of the blood parameters (*p* > 0.05). The differences in retinal artery parameters found between lean and overweight women did not change with increasing BMI values.

## 4. Discussion

In this study we provided noninvasive measurements of cones and retinal arteries morphologies with rtx1 AO in a group of healthy women. We excluded from the study women with already diagnosed hypertension, prediabetes, diabetes mellitus, or other cardiovascular disorders, which resulted in a smaller study group. We categorized the participants into two groups depending on BMI values. The control group consisted of lean women and the study (BMI) group consisted of women with BMI >25 kg/m^2^.

Overweight and obesity are known risk factors for the development of metabolic syndrome, diabetes, cardiovascular disorders, and ischaemic stroke [1–8, 24, 25]. The pathophysiological mechanism behind the relationship between obesity and microvascular dysfunction is probably multifactorial and has been analysed in several studies [6, 8, 9, 11, 12].

Our results showed that the mean lumen and total diameter of the analysed retinal arteries were not significantly different between the control and BMI groups. We found significant differences in the thickness of retinal arteriole walls in women with greater BMI. The WLR and WCSA parameters were also significantly higher in participants with overweight and obesity. Our results suggest that individuals with overweight and obesity demonstrate signs of an early dysfunction of the retinal arterioles as measured by the wall thickness and WLR parameters. These findings are in compliance with results described by de Jong and coworkers with a similar study group [8]. There were no differences in baseline perfused nail fold capillary density between lean and obese women, but postocclusive capillary recruitment and microvascular endothelium-dependent vasodilation were decreased in obese women [8]. Other studies have also demonstrated increased carotid intima-media thickness in young, healthy women, without known cardiovascular disease, but with obesity [7, 24, 25].

The obesity-related impairment in microvascular function may contribute to the increased risk of developing microangiopathy, hypertension, and insulin resistance [8, 9, 11, 26, 27].

Retinal circulation is closely related to cerebral circulation and may be a marker of cerebral arteriole health. There is some evidence that obesity, even in healthy individuals, is associated with an increased risk of ischaemic stroke [24, 25]. In a large prospective cohort study of 39,000 apparently healthy women, BMI was strongly associated with total and ischaemic stroke but not with haemorrhagic stroke [25]. A statistically significant trend across BMI categories for total and ischaemic stroke was found with increased risks for ischaemic stroke beginning with BMI values 27.0 kg/m^2^, which steadily increased with higher BMI. Women with BMI 35 kg/m^2^ had a 2-fold increase in the risk of total stroke and an almost 3-fold increase in the risk of ischaemic stroke compared with women with BMI 20 kg/m^2^ [25]. Data from the Nurses' Health Study showed similar associations and found a 2-fold increase in the risk of ischaemic stroke among women with BMI 32 kg/m^2^compared with women with BMI 21 kg/m^2^ [24].

In clinical practice, the examination of the retinal vessels is commonly used to assess retinal microvascular damage in diabetes and as an indicator of target organ damage in hypertension.

Although all individuals participating in our study were normotensive, systolic and diastolic blood pressure were significantly higher in women with higher BMI compared to the control group. The fact that there were some differences in blood pressure could explain the differences in microvascular function between lean and obese women. This may suggest that the presence of obesity is an important predictor of microvascular dysfunction before the onset of hypertension. These suggestions are consistent with the results of other authors who described the association between obesity and microvascular dysfunction [9–11].

Changes in small artery structure characterized by increased WLR are a characteristic feature of arterial remodelling in hypertension [28–30]. This finding can be the result of growth and/or remodelling of the vascular wall [28, 29]. In our study, we found increases of both parameters WLR and WCSA that may suggest growth of the arteries' wall.

We have described similar findings in retinal arteries in another study in patients with diagnosed prediabetes, without hypertension or other vascular disorders, where the increased WLR parameter was correlated with increased BMI and total cholesterol [31].

In contrast to our results are the results from the large prospective study of adults from Wisconsin, where retinal arteriolar narrowing was not found to be positively associated with obesity development during a 15-year follow-up [12]. Retinal venular widening has been identified among previously nonobese subjects who gained weight during the period of observation as significantly different than in participants with normal weight [12].

Aside from the assessment of retinal vessels, we used the rtx1 camera to analyse and compare cone density and regularity at different BMI levels. Our findings indicate that cone density and morphology are not affected by weight in healthy eyes.

### 4.1. Study Limitations

We have identified several possible limitations of this study, such as study size and inclusion criteria. Due to our inclusion criteria, we did not check the retinas of participants with diagnosed cardiovascular disorders. We also cannot be sure that none of the participants had insulin resistance because this was not analysed. The only participants of this study were women because we wanted to exclude sex differences in adipose tissue distribution and its possible influence on the vasculature. The study was designed as an exploratory study without an a priori sample size calculation. The present results may underestimate the effects of obesity in general because we studied a group of healthy, nonhypertensive, and nondiabetic overweight and obese women. Future prospective studies with an expanded sample, including both genders, should track changes in the microvascular and metabolic risk factors and explore their possible interactions.

## 5. Conclusions

Retinal image analysis with rtx1 offers a novel noninvasive measurement of early changes in the neural cells and retina vasculature that are not detectable on routine clinical examination. AO retinal imaging accurately identified retinal microvasculature and may serve as a promising, noninvasive screening tool for the early detection of microvascular complications. Our observations are consistent with the findings in other vascular beds (skin, cardiac, and cerebral microcirculation) showing a generalized adverse effect of overweight and obesity on microvascular function. Retinal arterioles may present early pathological changes, even without the diagnosis of metabolic syndrome or cardiovascular disorders in overweight and obese women.

## Figures and Tables

**Table 1:** Overall and group characteristics (*n* = 47).

	Total	BMI group	Control group	
	*n* (%)	*n* (%)	*n* (%)	
No. of subjects	47 (100)	28 (59.6)	19 (40.4)	

	Mean ± SD	Mean ± SD	Mean ± SD	*p* value
Age (years)	48.9 ± 8.1	45.1 ± 9.7	50.8 ± 6.4	0.065
AL (mm)	23.20 ± 1.03	23.21 ± 0.99	23.12 ± 0.97	0.974^†^
BMI	28.1 ± 4.9	31.1 ± 3.8	23.8 ± 1.2	<0.001

^†^ *t* test; SD: standard deviation; AL: mean axial length; BMI: body mass index.

**Table 2:** The results of the blood parameters in both groups (*n* = 47) after Bonferroni correction (adjusted *p* value = 0.0071).

Blood parameters	Mean ± SD	Mean ± SD	*p* value
	Control group	BMI group	
WBC (k)	6.56 ± 1.81	6.60 ± 1.69	0.839
RBC (M)	4.66 ± 0.35	4.55 ± 0.26	0.444
HGB (g/dl)	13.21 ± 1.37	13.46 ± 0.90	0.457
HCT (%)	40.56 ± 3.18	40.62 ± 2.30	0.991
PLT (k)	266.2 ± 77.4	252.0 ± 58.3	0.482
FPG (mg/dl)	92.6 ± 7.2	96.9 ± 7.3	0.028
HBA_1C_ (%)	5.36 ± 0.31	5.5 ± 0.31	0.333

SD: standard deviation; FPG: fasting plasma glucose; HbA1c: glycated haemoglobin A1c; TG: triglyceride; LDL: low-density lipoprotein cholesterol; HDL: high-density lipoprotein cholesterol; WBC: white blood cells; RBC: red blood cells; HGB: haemoglobin; HCT: haematocrit; PLT: platelets.

**Table 3:** The results of the serum lipid parameters in both groups (*n* = 47) after Bonferroni correction (adjusted *p* value = 0.0125).

Serum lipid parameters	Mean ± SD:	Mean ± SD:	*p* value
	Control group	BMI group	
Total cholesterol (mg/dl)	198.8 ± 28.8	189.8 ± 21.4	0.229
HDL (mg/dl)	64.1 ± 12.5	53.9 ± 12.9	0.012^*∗*^
LDL (mg/dl)	112.5 ± 23.9	125.8 ± 30.1	0.120
TG (mg/dl)	108.9 ± 61.0	136.6 ± 33.0	0.006^*∗*^

SD: standard deviation; TG: triglyceride; LDL: low-density lipoprotein cholesterol; HDL: high-density lipoprotein cholesterol; WBC: white blood cells; RBC: red blood cells; HGB: haemoglobin; HCT: haematocrit; PLT: platelets. ^*∗*^Statistically significant results after Bonferroni correction (*p* < 0.0125).

**Table 4:** Mean cone density in 4 retinal quadrants at 2°eccentricities in both groups (*n* = 47) after Bonferroni correction (adjusted *p* value = 0.0125).

Quadrants	Mean (±SD) cone density, cone/mm^2^	Mean (±SD) cone density, cone/mm^2^	*p* value^†^
	Control group	BMI group	
*T*	26478 ± 2211	24144 ± 2654	0.006^*∗*^
*N*	23880 ± 6924	24597 ± 2265	0.622
*S*	23694 ± 7077	23734 ± 2874	0.979
*I*	23262 ± 7064	23879 ± 2415	0.679

^†^ *p* t test; SD:, standard deviation; T: temporal; N: nasal; S: superior; I: inferior. ^*∗*^Statistically significant results after Bonferroni correction (*p* < 0.0125).

**Table 5:** Mean (±SD) spacing, Voronoi, and cone regularity in Control and BMI groups in 4 quadrants after Bonferroni correction (adjusted *p* value = 0.0042).

Cone parameters in 4 quadrants	Control group	BMI group	*p* value
Spacing_*T* (*μ*m)	6.80 ± 0.30	7.13 ± 0.38	0.006
Voronoi *N*%6_*T* (%)	47.7 ± 5.9	45.2 ± 5.1	0.154
Regularity_*T* (%)	93.9 ± 3.2	93.1 ± 2.7	0.368
Spacing_*N* (*μ*m)	6.45 ± 1.81	7.06 ± 0.32	0.096
Voronoi *N*%6_*N* (%)	44.2 ± 12.8	47.3 ± 5.4	0.280
Regularity_*N* (%)	88.2 ± 24.5	93.9 ± 2.4	0.228
Spacing_*S* (*μ*m)	6.48 ± 1.83	7.19 ± 0.44	0.058
Voronoi *N*%6_*S* (%)	44.4 ± 13.3	48.4 ± 5.7	0.1135
Regularity_*S* (%)	71.5 ± 18.9	75.6 ± 21.2	0.4949
Spacing_*I* (*μ*m)	7.46 ± 2.06	6.57 ± 1.86	0.1586
Voronoi *N*%6_*I* (%)	41.7 ± 11.8	44.4 ± 13.3	0.4866
Regularity_*I* (%)	86.5 ± 22.3	88 ± 24.5	0.8371

SD: standard deviation; *T*: temporal; *N*: nasal; *S*: superior; *I*: inferior; regularity, the summary of the percentage of pentagonal, hexagonal, and heptagonal cones in the analysed image; Voronoi *N*%6: percentage of hexagonal cones in analysed image; spacing, interphotoreceptor distance.

**Table 6:** Characteristic of retinal artery parameters in the BMI and control groups (*n* = 28 and *n* = 19) after Bonferroni correction (adjusted *p* value = 0.0084).

Parameters	Mean (±SD)	Mean (±SD)	*p* value
	Control group	BMI group	
Lumen (*μ*m)	96.47 ± 12.20	99.97 ± 11.30	0.352†
Total diameter (*μ*m)	120.5 ± 14.4	128.8 ± 13.3	0.064†
WALL_1 (*μ*m)	12.14 ± 1.9	14.21 ± 2.7	<0.001†^*∗*^
WALL_2 (*μ*m)	11.06 ± 3.6	14.60 ± 2.62	<0.001^*∗*^
WLR (1)	0.25 ± 0.03	0.29 ± 0.03	<0.001†^*∗*^
WCSA (*μ*m^2^)	4136.7 ± 961.0	5217.3 ± 1041.0	<0.001^*∗*^

^†^ *t* test; SD: standard deviation; WLR: wall-to-lumen ratio; WCSA: wall cross-sectional area.  ^*∗*^Statistically significant results after Bonferroni correction (*p* < 0.0084).

## Data Availability

The data sets used and analysed during this study are available from the corresponding author in consideration of potentially applying restrictions on reasonable request.
